# Indium‐Mediated Glue‐Like Interlayer Enables Stable High‐Capacity Flexible Sodium Metal Batteries

**DOI:** 10.1002/adma.73556

**Published:** 2026-05-29

**Authors:** Xinyan Li, Shujing Wen, Junhua Zhou, Yanpeng Guo, Jiehua Cai, Xingyang Wang, Yongqiang Yang, Ang Ye, Can Guo, Zhaokun Wang, Qiyao Huang, M. Danny Gu, John Wang, Zijian Zheng

**Affiliations:** ^1^ Department of Applied Biology and Chemical Technology, Faculty of Science The Hong Kong Polytechnic University Hung Hom Hong Kong SAR China; ^2^ Department of Materials Science and Engineering National University of Singapore Singapore Republic of Singapore; ^3^ School of Fashion and Textiles The Hong Kong Polytechnic University Hong Kong SAR China; ^4^ Institute for Advanced Study Eastern Institute of Technology Ningbo Zhejiang China; ^5^ Research Institute for Smart Energy The Hong Kong Polytechnic University Hung Hom Hong Kong SAR China; ^6^ Research Institute for Intelligent Wearable Systems The Hong Kong Polytechnic University Hung Hom Hong Kong SAR China; ^7^ PolyU‐Daya Bay Technology and Innovation Research Institute Huizhou Guangdong China; ^8^ PolyU‐Wenzhou Technology and Innovation Research Institute Wenzhou Zhejiang China

**Keywords:** flexible battery, high‐loading cathode, interface, metal anode, sodium battery

## Abstract

Na metal is considered as a highly promising anode material for Na batteries, owing to its high theoretical capacity (1166 mAh g^−1^) and low redox potential (−2.71 V vs. SHE). However, its practical application is significantly hindered by interfacial instability and dendrite formation arising from uneven Na deposition, which severely compromise its cycling lifespan. Herein, we develop a robust three‐dimensional glue‐like interlayer via in situ electrochemical conversion of CuInS_2_ on a flexible Cu current collector. This process yields a Na_2_S/Na_5_InS_4_/Cu composite interlayer of strong interphase connections, ensuring tight interfacial connections and exceptional mechanical stability throughout extended cycling, thereby effectively mitigating interfacial degradation. With this interlayer, the Na metal anode achieves a high Coulombic efficiency over 99.4%, outstanding symmetrical cell stability exceeding 2400 hours, and excellent high‐capacity full cell cycling (95.3% capacity retention after 1000 cycles). Flexible pouch cells retain 95% capacity over 200 cycles and endure more than 10,000 bending.

## Graphical abstract

A three‐dimensional Na‐In‐S/Cu composite interlayer is constructed on a flexible current collector via an In‐mediated in situ reaction. Featuring abundant Na nucleation sites and a low diffusion barrier, this interlayer enables rapid and uniform Na deposition. Notably, the in situ generated Na_5_InS_4_ exhibits strong binding with both the Cu and Na_2_S phases, creating a unique glue‐like effect that ensures tight interphase connections and mechanical stability during long‐term cycling. Benefiting from this robust interlayer, the electrode accommodates high‐mass‐loading cathodes and demonstrates excellent cycling stability and flexibility in pouch cells.

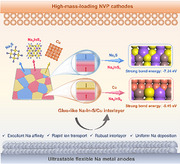

## 1 Introduction

Na metal batteries (SMBs) receive increasing attention nowadays, because of the natural abundance of Na resources, and the low redox potential (−2.71 V vs. SHE) and high theoretical capacity (1166 mAh g^−1^) of Na metal [1]. These features render SMBs particularly attractive for next‐generation large‐scale grid energy storage [2, 3]. However, practical application of Na metal anodes remains challenging, rooted in the complex solid‐liquid interfacial behavior during Na plating and stripping [4]. Uneven Na deposition induces uncontrolled dendrite growth that may penetrate the separator and subsequently lead to internal short circuits [5]. In the meanwhile, the formation of electrochemically inactive “dead Na” further deteriorates the Coulombic efficiency (CE) and the cycling stability [6]. Moreover, repeated volume changes of the electrode during cycling induce continuous mechanical stress and strain on the solid electrolyte interphase (SEI) [7], which leads to its rupture [8] and subsequent exposure of fresh electrode surfaces to the electrolyte. This consumes a large amount of electrolyte due to its decomposition and the repeated SEI reformation, and ultimately imposes severe limitations on the cycling lifespan of SMBs [9, 10].

To overcome the persistent challenges associated with Na metal anodes, several strategies including electrolyte engineering [11], construction of artificial interfacial layer [12, 13, 14], and design of three‐dimensional (3D) conductive framework, have been reported to date [15]. Among these, 3D current collectors such as Cu foam, Ni foam, and carbon cloth are particularly attractive, as their large surface areas can effectively reduce the local current density and accommodate volume changes to suppress dendrite formation [16]. However, these 3D scaffolds are inherently sodiophobic, which result in poor Na affinity, high nucleation barriers, and uneven Na deposition [17].

To improve the interfacial wettability, researchers introduced sodiophilic interlayer coatings such as Sn, Bi, In or Sb onto 3D frameworks to reduce the nucleation overpotential by leveraging their alloying reactions with Na [9, 18]. While such alloying layers can improve the wettability of the anode toward Na, the substantial volume expansion associated with these reactions often undermines the structural integrity of the coating [19]. Over prolonged cycling, these interlayers suffer from exceptional internal stress, that are susceptible to cracking and even delamination from the substrate [20, 21]. Moreover, dense sodiophilic coatings can impede rapid Na^+^ diffusion, leading to non‐uniform deposition, dendrite growth, and exacerbated interfacial side reactions [22]. Collectively, these limitations diminish the effectiveness of sodiophilic interlayer coatings in regulating Na plating and stripping. Therefore, to fundamentally overcome interfacial failure and to achieve highly reversible, dendrite‐free SMBs, it is crucial to construct a robust and long‐term stable interlayer that preserves structural integrity and sustained Na affinity, while simultaneously enabling rapid Na^+^ transport over prolonged cycling [23].

Herein, we report an in situ conversion strategy for constructing a sodiophilic composite interlayer, which leverages a unique “glue‐like” effect to sustain long‐term structural integrity. Our approach starts with the direct growth of 3D CuInS_2_ nanostructures on a flexible Cu current collector. The CuInS_2_ layer is then electrochemically converted into a composite interlayer composed of Na_2_S, Na_5_InS_4_, and Cu (denoted as Na‐In‐S/Cu) via discharge of Na^+^ ions. This interlayer not only possesses abundant Na nucleation sites and low diffusion barriers, but also exhibits a unique “glue‐like” effect, which stems from the strong internal cohesion within the Na‐In‐S/Cu composite interlayer itself. Specifically, robust interactions between the in situ formed Na_5_InS_4_ with both the Cu and Na_2_S phases integrate these components, ensuring mechanical stability. Such internal cohesion ensures tight interphase connections during long‐term cycling, thereby imparting excellent mechanical stability to the interface. Furthermore, this stable interlayer induces the formation of a thin, dense, and mechanically robust SEI, which effectively accommodates electrode volume fluctuations, suppresses dendrite growth and significantly enhances the overall electrochemical stability of the interface. Consequently, Na anode achieves a high CE (>99.4%) over 500 cycles. Symmetrical cells remain stable for over 2400 hours at 1 mA cm^−2^. SMBs equipped with high‐loading Na_3_V_2_(PO_4_)_3_ (NVP) cathodes (10.2 mg cm^−2^) retain 95.3% capacity after 1000 cycles, and 95.5% after 120 cycles even at an ultrahigh loading of 26.7 mg cm^−2^. Importantly, flexible pouch cells retain 95% capacity over 200 cycles and endure 11,000 bending cycles, demonstrating great potential for flexible Na batteries.

## 2 Results and Discussion

### 2.1 Design of 3D Glue‐Like effect Na‐In‐S/Cu Interlayer

**FIGURE 1:**
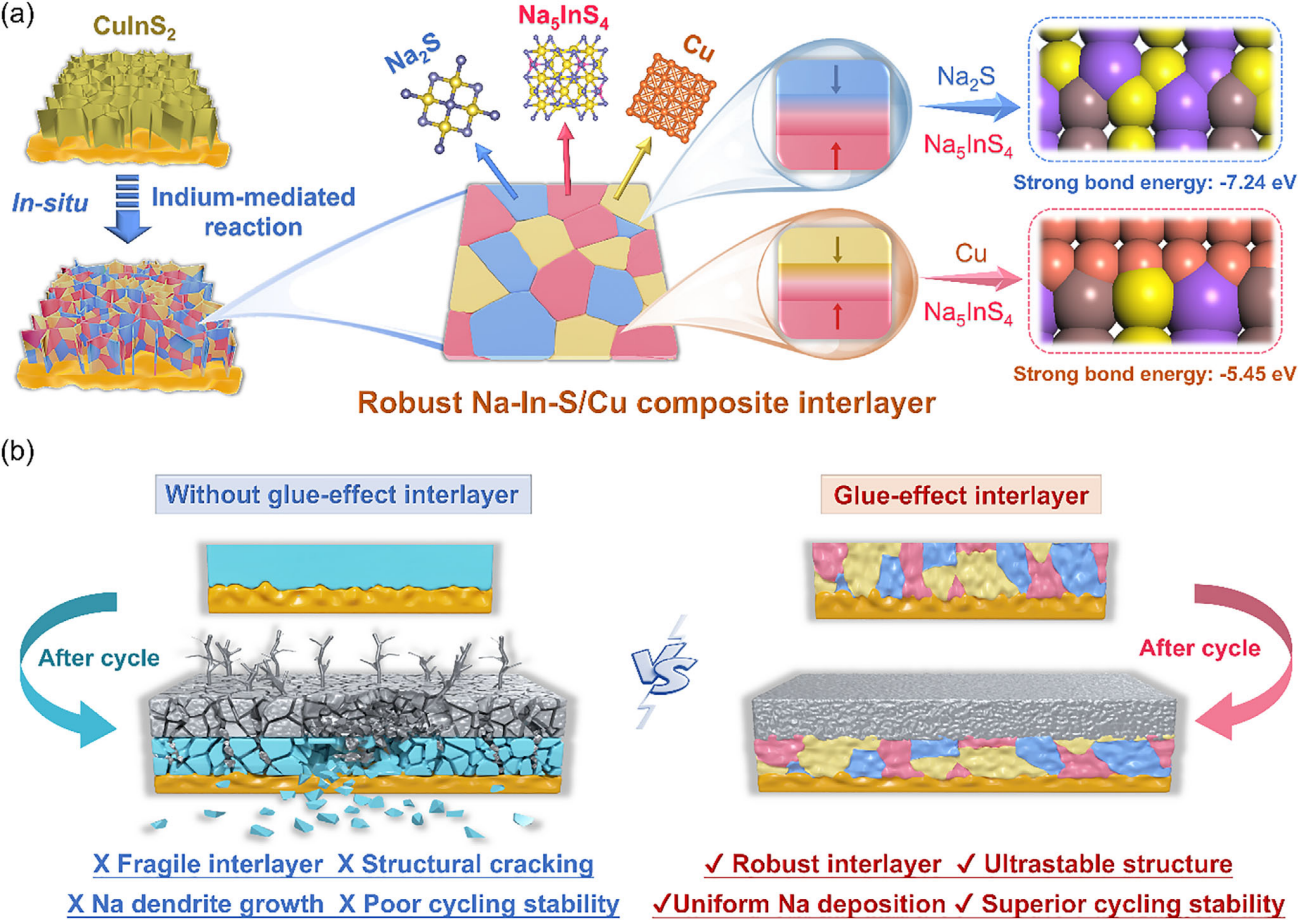
Design concept and advantages of a stable interlayer. (a) Schematic illustration of a 3D glue‐like effect composite interlayer via an In‐mediated in situ reaction. The Na_5_InS_4_ phase serves as a glue, tightly integrating the interface components and providing excellent mechanical stability to accommodate volume and stress changes during Na cycling. (b) Comparison of interlayer characteristics: the in situ formed interlayer (right) exhibits tight interfacial connections and robust mechanical stability, and enables uniform Na plating, while the conventional artificial interlayer (left) displays weak adhesion, structural cracking, detachment and Na dendrite growth, leading to rapid structural degradation.

Figure 1a illustrates the core concept and key advantages of the glue‐like effect of the stable interlayer. Utilizing an In‐mediated in situ reaction strategy, a 3D glue‐like effect interlayer is constructed on an ultralight and flexible current collector. During the initial discharge, CuInS_2_ nanowalls are directly converted into a composite interlayer consisting of Na‐In‐S/Cu. Notably, the Na_5_InS_4_ phase acts as a “glue”, tightly integrating the different components and significantly enhancing the mechanical stability of the interlayer. This robust cohesion enables the interlayer to effectively accommodate volume changes and bending stresses during Na plating and stripping, thereby imparting excellent ultrastability. Figure 1b further compares the structural integrity of electrodes with and without the glue‐like effect interlayer. As shown in the panel on the right hand side, the in situ formed interlayer exhibits strong adhesion to the substrate, and its 3D glue‐like structure maintains interfacial integrity throughout cycling. This design effectively suppresses crack formation and Na dendrite growth, while promotes uniform Na deposition. In contrast, conventional artificial interlayer (panel on the left hand side) exhibits weak adhesion to the substrate and lack internal binding mechanisms, resulting in structural cracking, detachment, and uncontrolled Na dendrite growth, these shortcomings ultimately lead to rapid interfacial failure and impossible to achieve long‐term cycling stability [24].

### 2.2 Fabrication and Characterizations of 3D Glue‐Like Na‐In‐S/Cu Interlayer

**FIGURE 2:**
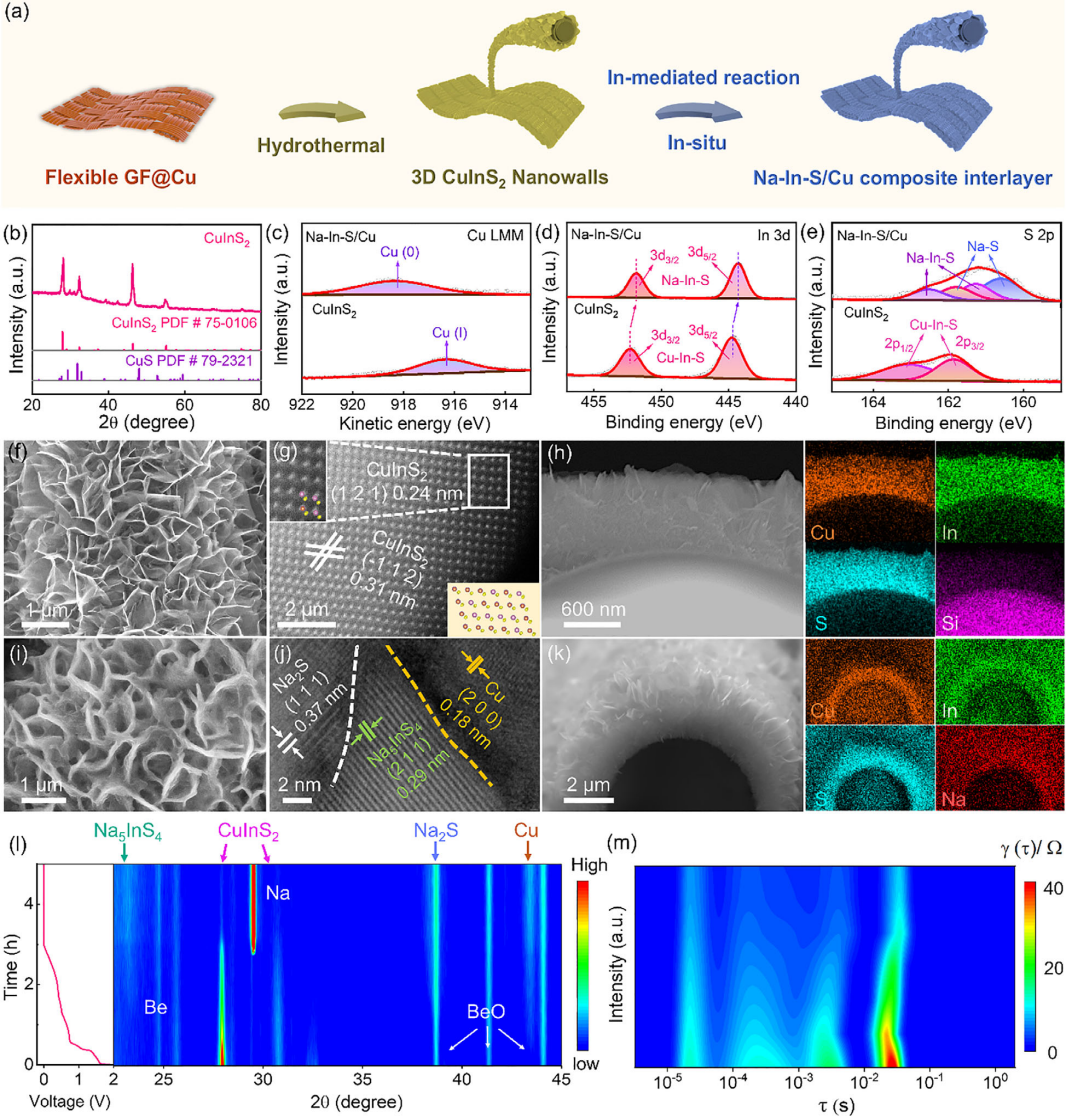
Synthesis and characterizations of the CuInS_2_ nanowalls and sodiophilic Na‐In‐S/Cu composite interlayer. (a) Schematic illustration of the synthesis process. (b) XRD pattern of CuInS_2_ nanowalls (PDF #75–0106). (c) XPS core‐level spectra of Cu LMM, (d) In 3d and (e) S 2p. (f) SEM top‐view images of CuInS_2_. (g) STEM image of the CuInS_2_ atomic structure. The insets show the corresponding atomic models with yellow sphere as S, rose red as In and orange sphere as Cu. (h) SEM side‐view and corresponding elemental mapping of CuInS_2_. (i) SEM images of Na‐In‐S/Cu composite interlayer. (j) HRTEM images indicating the presence of Na_2_S, Na_5_InS_4_ and Cu. (k) SEM side‐view and corresponding elemental mapping of Na‐In‐S/Cu composite interlayer. (l) In situ XRD patterns of Na conversion and plating process on CuInS_2_ during the first discharge. (m) DRT contour map of Na conversion during the first discharge.

Figure 2a illustrates the preparation approach for the interlayer, which includes three major steps: 1) deposition of Cu onto glass fibers (GF), 2) formation of CuInS_2_ nanoarrays on Cu via a hydrothermal process and 3) in situ conversion of CuInS_2_ into the glue‐like composite interlayer Na‐In‐S/Cu. Lightweight GF fabric (30 µm, 2.3 mg cm^−2^) was selected as the flexible substrate and coated with a Cu layer via a polymer‐assisted metal deposition method [25]. This process yielded an ultralight GF@Cu current collector, whose total mass (3.1 mg cm^−2^) is significantly lower than that of conventional commercial Al foil (4.2 mg cm^−2^), Cu foil (7.5 mg cm^−2^) and flexible carbon cloth (12.5 mg cm^−2^). A uniform Cu coating on the GF surface was confirmed by scanning electron microscopy (SEM) and energy dispersive X‐ray spectroscopy (EDS), where the Si signal arises from the GF substrate (Figure S1).

We first grew Cu‐In‐S on the Cu surface via a hydrothermal process followed by a mild thermal annealing. X‐ray diffraction (XRD) analysis reveals that the product predominantly consists of the CuInS_2_ phase (PDF #75–0106) with minor CuS phase (PDF #79–2321) (Figure 2b). For CuInS_2_, the Cu 2p and Cu LMM Auger spectra confirm the predominant presence of Cu^+^ (Figure 2c and Figure S2), the In 3d peaks at 452.3 and 444.7 eV correspond to In^3+^ (Figure 2d), and S 2p doublet is characteristic of S^2−^ (Figure 2e) (X‐ray photoelectron spectroscopy (XPS) [26, 27]. Morphologically, the as‐prepared CuInS_2_ exhibits a nanowalls architecture, which is uniform distributed on each fiber and interconnect to form a continuous 3D porous network (Figure 2f and Figure S3a–d). This well‐organized structure is further corroborated by transmission electron microscopy (TEM, Figure S3e–h), and reveals lattice fringes with a spacing of 0.31 nm corresponding to the (112) plane of the CuInS_2_ (Figure S4a), while confirm a polycrystalline nature (Figure S4b). High‐angle annular dark‐field scanning TEM image further elucidates atomic arrangements consistent with the CuInS_2_ phase (Figure 2g). In addition, these uniquely structured nanowalls are closely integrated with the current collector, and the elements Cu, In, and S are uniformly distributed throughout the entire structure uniform distribution of Cu, In, and S (Figure 2h).

We then converted the CuInS_2_ layer into the targeted glue‐like Na‐In‐S/Cu composite interlayer via an in situ conversion reaction during the initial discharge process. The Cu 2p spectra (Figure S2) and Cu LMM Auger spectroscopy were used to confirm the reduction of Cu(I) in pristine CuInS_2_ to metallic Cu(0) upon interlayer formation (Figure 2c) [28]. The In 3d spectra show a shift toward lower binding energy (Figure 2d), indicating a change in the coordination environment from Cu‐In‐S to Na‐In‐S [29]. Additionally, the S 2p spectra reveal the emergence of two new sulfur species (Figure 2e), assigned to Na‐In‐S and Na‐S [30]. The coexistence of these phases suggests that Na_5_InS_4_ plays a role in interfacial evolution, involving Na_2_S‐related sulfide chemistry. Furthermore, after this transformation, the interlayer retains a robust 3D nanowalls architecture, and the nanowalls become significantly thicker and structurally reinforced (Figure 2i). This architecture is expected to facilitate electrolyte infiltration and effectively accommodates volume changes during cycling [19]. The presence of distinct lattice fringes about 0.18 nm corresponding to the (200) plane of Cu, 0.29 nm to the (211) plane of phase Na_5_InS_4_, and 0.37 nm to the (111) plane of phase Na_2_S (Figure 2j), confirms the formation of the desired composite phases. In addition, the uniform distribution of Na, In, S, and Cu elements throughout the interlayer demonstrates the successful construction of a homogeneous composite structure (Figure 2k).

To elucidate the dynamic formation mechanism of the interlayer, the evolution of phase composition during the initial discharge process was tracked in real time by in situ XRD (Figure 2l). During this process, the gradual disappearance of CuInS_2_ is accompanied by the emergence of Cu, Na_5_InS_4_ and Na_2_S, indicating that CuInS_2_ undergoes a conversion reaction to yield a composite interlayer composed of these phases. This transformation is consistent with the structural features observed at the nanoscale, collectively confirming the successful formation of the targeted composite interlayer. Meanwhile, a continuous decrease in interfacial resistance is observed during discharge *by* in situ electrochemical impedance spectroscopy (EIS) (Figure 2m and Figure S5), which correlates closely with the formation of the aforementioned stable interlayer. This reduction in interfacial resistance indicates that the composite interlayer possesses excellent mixed ionic/electronic conductivity [31], thereby significantly lowering the interfacial charge‐transfer impedance and establishing a robust foundation for subsequent efficient and uniform Na plating and stripping. By setting the charge cut‐off voltage at 0.3 V, Na^+^ extraction is effectively suppressed, maintaining the structural integrity of the interlayer throughout cycling. As shown in Figure S6, no obvious oxidation peaks are observed within the 0–0.3 V range, indicating the negligible Na^+^ extraction in this range. Additionally, the capacity‐potential profiles exhibit no reaction plateau during charging, further confirming that the conversion is confined to the initial cycle (Figures S7 and S8). This ensures that subsequent Na plating/stripping occurs exclusively on the stable, in situ formed composite interlayer. The sustained presence of characteristic diffraction peaks in in situ XRD throughout subsequent cycles further demonstrates the chemical and structural robustness of this composite interlayer (Figure S9).

### 2.3 Theoretical Investigation of Na‐In‐S/Cu Interlayer via DFT and FEA

**FIGURE 3:**
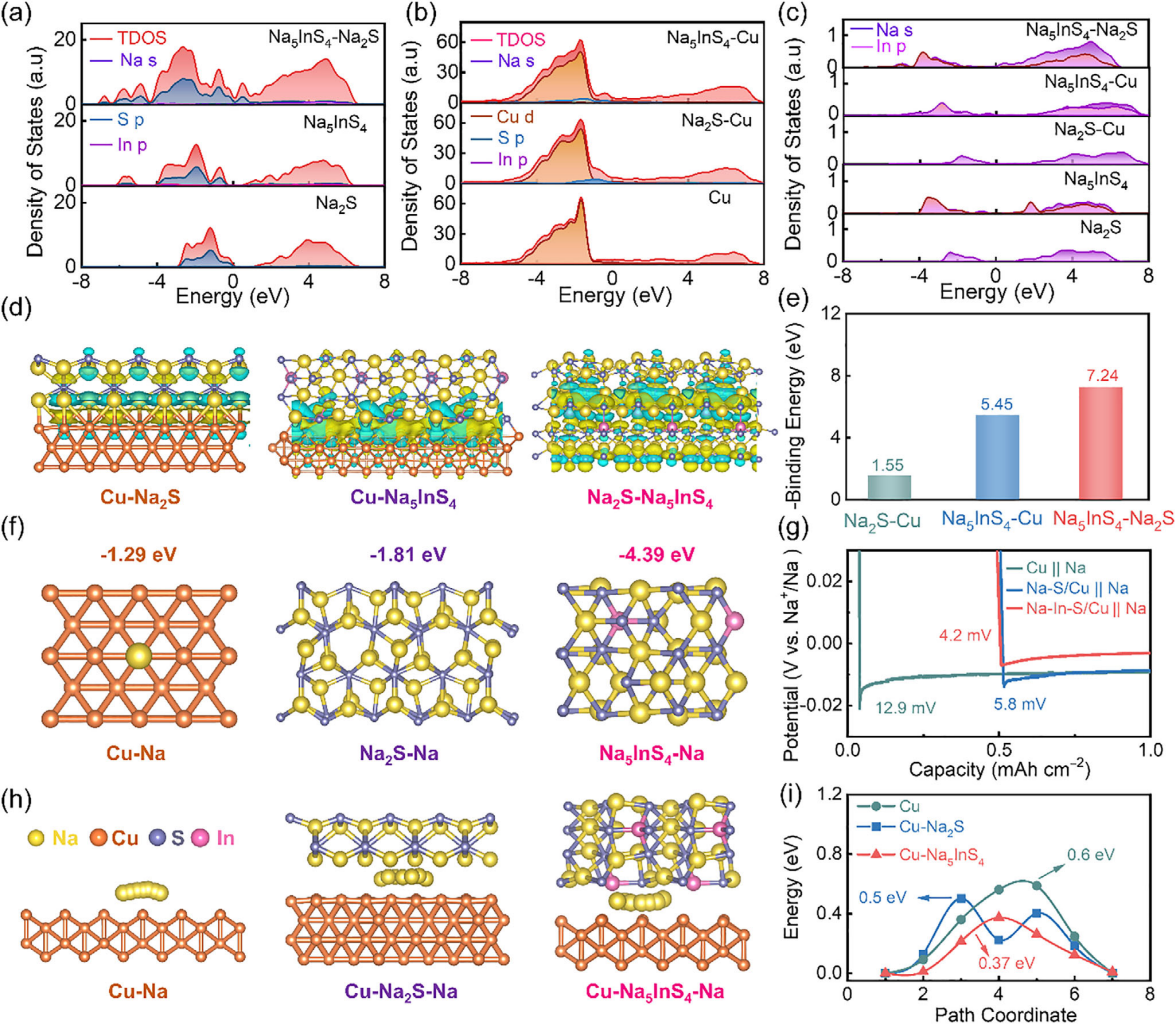
Theoretical calculation based on DFT of composite interlayers. (a) DOS of Na_5_InS_4_‐Na_2_S, Na_5_InS_4_, and Na_2_S. (b) DOS of Na_5_InS_4_‐Cu, Cu and Na2S‐Cu. (c) Magnified views of the Na and In from the Figures a) and b). (d) Charge density difference of the three interface models: Cu‐Na_2_S, Cu‐Na_5_InS_4_, and Na_2_S‐Na_5_InS_4_. The blue and yellow regions represent positive and negative charge differences, respectively. (e) Corresponding binding energy for the models in d). (f) Geometrically optimized models and corresponding adsorption energy among Cu, Na_2_S, Na_5_InS_4_ with Na. (g) Initial capacity‐voltage curves of half‐cells tested at 1 mA cm^−2^ for 1 mAh cm^−2^. (h) Na migration pathways and i) diffusion energy barriers in the Cu, Cu‐Na_2_S, and Cu‐Na_5_InS_4_ models.

The stability of the composite interlayer was assessed via density functional theory (DFT) calculations. Interface models of Na_5_InS_4_‐Na_2_S, Na_5_InS_4_‐Cu, and Na_2_S‐Cu were constructed, and their corresponding density of states (DOS) were compared with those of the individual phases Na_5_InS_4_, Na_2_S, and Cu (Figure 3a–c). For the Na_5_InS_4_‐Na_2_S interface, the DOS reveals the emergence of new states and an enhanced intensity near the Fermi level compared to the individual phase (Figure 3a). This indicates an increase in the density of electronically active states, which facilitates interfacial charge redistribution and promotes more efficient charge exchange between the two sulfides [32]. The observed peak broadening further suggests strengthened orbital hybridization [33, 34]. For the Na_5_InS_4_‐Cu interface, the DOS broadening is more pronounced compared to both Na_2_S‐Cu and Cu, indicating enhanced orbital hybridization and stronger electronic coupling between Na_5_InS_4_ and Cu (Figure 3b) [35]. These characteristics imply that Na_5_InS_4_ can effectively mediate electronic interaction between the sulfide and metallic phases. The interfacial coupling behavior provides a potential basis for understanding the proposed “glue‐like” interfacial stabilization mechanism.

Differential charge density analysis offers further insight into the interfacial stabilization mechanism at the electronic level, revealing obvious electron accumulation at the interface (Figure 3d). A higher degree of electron accumulation typically associated with stronger bonding interactions and enhanced interfacial stability [36]. To quantitatively assess interfacial stability from an energy perspective, we calculated the binding energy between different components (Figure 3e). The binding energy between Na_2_S and Cu is −1.55 eV, notably, the introduction of the Na_5_InS_4_ phase significantly increases the binding energy with both Cu (−5.45 eV) and Na_2_S (−7.24 eV). These results suggest that the Na_5_InS_4_ phase acts as a “interfacial glue” within the composite interface, effectively enhancing the interface binding strength, thereby providing reliable mechanical support for stable Na metal plating and stripping [37].

Beyond structural robustness, favorable sodiophilicity at the interlayer is essential for optimal Na metal anode performance. The adsorption energies of Na atoms on various interfacial components were evaluated to quantify their sodiophilic characteristics. For the initial components, Na affinity increases from Cu to CuS and further to CuInS_2_ (Figure 3f and Figure S10), indicating that sulfide incorporation effectively enhances the intrinsic sodiophilicity. Upon the formation of the Na‐In‐S/Cu interlayer, Na_5_InS_4_ exhibits more negative adsorption energy (−4.39 eV) than pure Cu (−1.29 eV) and Na_2_S (−1.81 eV), reflecting a much stronger affinity for Na (Figure 3f) [38]. This enhanced adsorption is expected to lower the nucleation barrier for Na plating and increase the number of active sites for uniform nucleation and deposition [39]. These theoretical predictions are corroborated by experimental Na deposition studies. As shown in Figure 3g, the pure Cu exhibits the highest nucleation overpotential (12.9 mV), which decreases to 5.8 mV with the introduction of CuS to form Na_2_S and Cu interlayer (denoted as Na‐S/Cu), and further to 4.2 mV with the Na‐In‐S/Cu composite interlayer. These results confirm that the In‐mediated composite interlayer effectively reduces the nucleation barrier for Na, consistent with the DFT calculated adsorption energy trends.

Notably, while strong adsorption is essential for an ideal Na metal anode, interfacial Na ion diffusion is equally critical, as it directly impacts deposition uniformity and dendrite suppression. Diffusion energy barriers for Na across different interfacial models were calculated (Figure 3h,i), revealing that the interlayer containing Na_5_InS_4_ exhibits the lowest diffusion barrier (0.37 eV), enabling rapid Na migration [40]. This efficient ion transport promotes uniform Na ion flux redistribution, alleviates local concentration polarization, and effectively suppresses Na dendrite growth [41].

**FIGURE 4:**
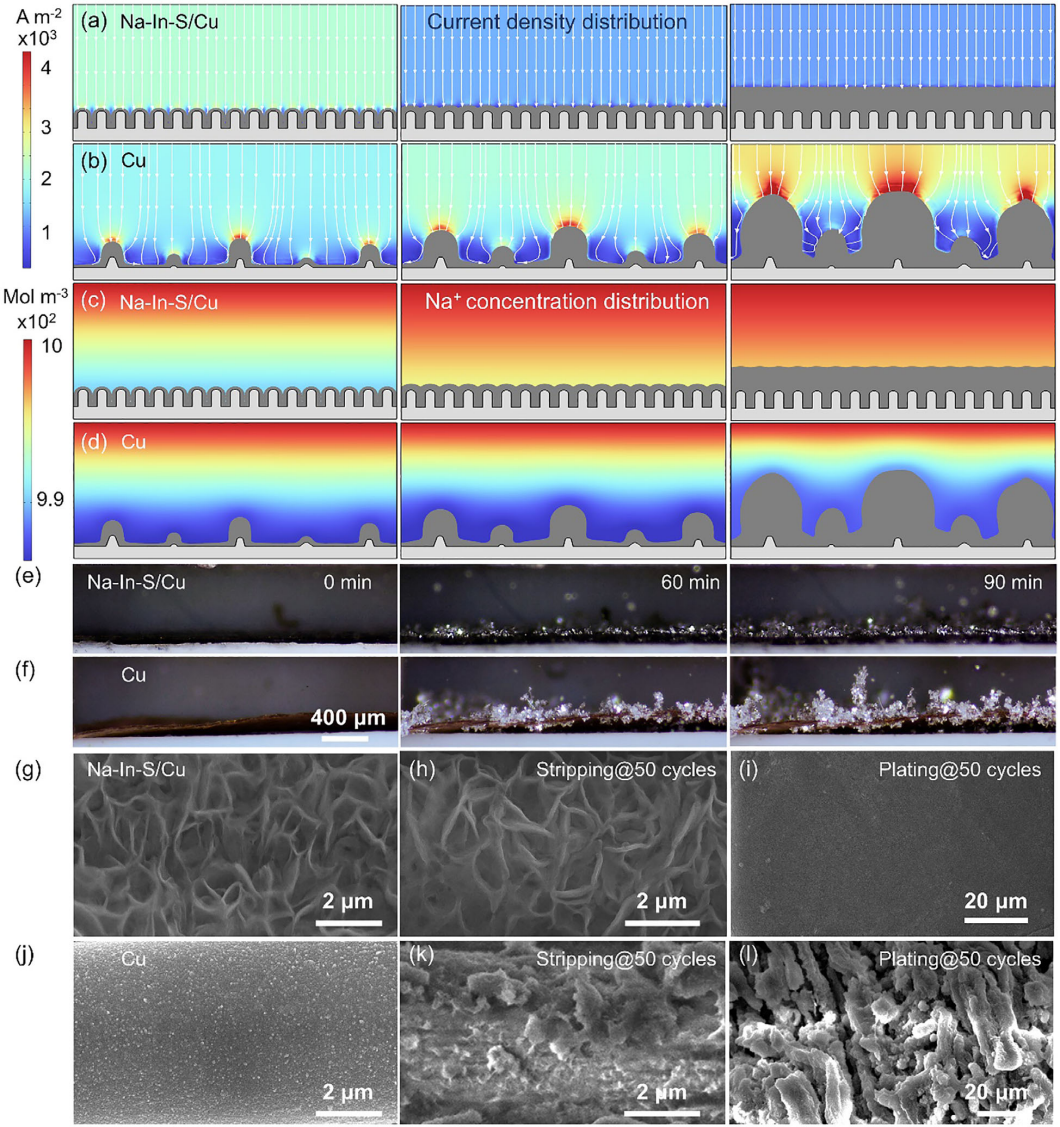
Integrated analysis of current density, Na^+^ concentration, morphology for the electrode featuring Cu and Na‐In‐S/Cu in Na metal batteries. Simulation results for Na‐In‐S/Cu and Cu, (a, b) Current density distribution. (c, d) Na^+^ concentration distribution. In situ optical detection of Na plating of (e) Na‐In‐S/Cu composite interlayer and (f) Cu at 2 mA cm^−2^. (g) SEM images of the Na‐In‐S/Cu composite interlayer after 5 cycles and (h) after 50 cycles. (i) SEM images of the Na‐In‐S/Cu composite after Na plating. (j) SEM images of the Cu and (k) after 50 cycles. (l) SEM images of the Cu after Na plating.

Atomic scale calculations demonstrate that the composite interlayer possesses excellent structural stability, strong Na affinity, and a low ionic diffusion energy barrier. To correlate these properties with uniform Na deposition, finite element analysis was conducted to simulate the evolution of electric field distribution, current density, and Na^+^ concentration near the interface (Figure 4a–d and Figure S11). Simulation results reveal that as Na deposition progresses, protrusions form on the surface of bare Cu, causing significant local distortion and enhancement of the electric field [22]. This intensified field at the electrolyte/anode interface leads to nonuniform Na^+^ diffusion and substantial Na^+^ accumulation at these protrusions, ultimately triggering dendrite growth [42]. In contrast, the electrode featuring Na‐In‐S/Cu interlayer maintains a uniform surface electric field and reduced local current density, attributable to the large surface area of nanowalls architecture. This enables homogeneous Na^+^ flux and uniform Na nucleation and deposition over time [43]. Furthermore, the abundant nucleation sites provided by the sodiophilic surface further enhance deposition uniformity.

To experimentally validate the above simulation results, we investigated the morphology of Na deposition on different substrates using SEM. On bare Cu, the deposited Na layer exhibited a loose, porous moss‐like structure with obvious roughness and non‐uniformity (Figure S12). Introducing a Na‐S/Cu interlayer moderately improved deposition uniformity, with no obvious dendrite formation observed (Figure S13). Notably, modification with the Na‐In‐S/Cu composition interlayer resulted in a significantly denser, smoother, and more uniformly distributed Na metal layer, indicating that Na‐In‐S/Cu composition interlayer effectively guides the homogeneous nucleation and growth of Na (Figure S14). Moreover, in situ deposition experiments using optical microscopy further confirmed that Na‐In‐S/Cu composition interlayer surface remains smooth and flat, with no observable Na dendrite formation (Figure 4e). In contrast, the bare Cu surface rapidly develops irregular protrusions and dendrites, which become increasingly prominent and cover the surface after 90 minutes (Figure 4f). Additionally, post‐cycling SEM revealed that the electrode featuring Na‐In‐S/Cu composition interlayer retains its intact 3D morphology without noticeable structural degradation (Figure 4g–i and Figure S15a). Even after 50 cycles, Na plating remains smooth and uniform, indicating high interfacial stability and highlighting the beneficial role of the Na‐In‐S/Cu interlayer in maintaining structural integrity. In contrast, the Cu exhibits the formation of dead Na, aggregation, and disordered dendritic growth, primarily resulting from uneven Na plating/stripping behavior (Figure 4j–l and Figure S15b). Collectively, these results demonstrate that the Na‐In‐S/Cu composition interlayer, benefiting from its uniform electric field distribution, abundant nucleation sites, and excellent ionic transport, markedly improves the uniformity of Na deposition, effectively suppresses dendrite growth, and provides a robust interfacial foundation for efficient Na cycling.

### 2.4 SEI Regulation via the Na‐In‐S/Cu Interlayer

**FIGURE 5:**
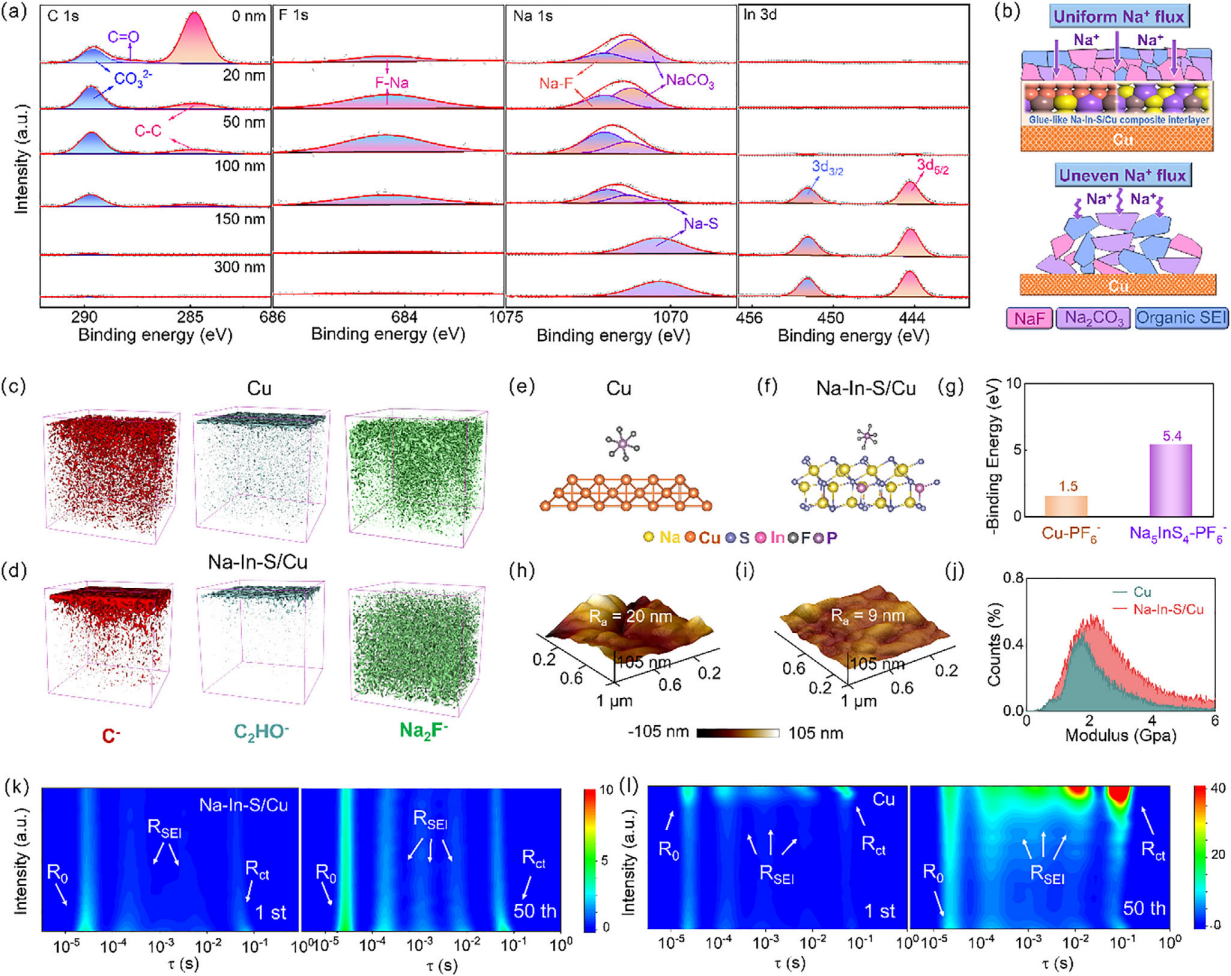
SEI component analysis. (a) High resolution XPS spectra of C 1s, F 1s, Na 1s and In 3d at specific sputtering time points after cycling of the electrode featuring Na‐In‐S/Cu interlayer. (b) Schematic illustration of the components and structure of the SEI. (c) the corresponding TOF‐SIMS 3D spatial distribution of representative ion fragments for the electrode without interlayer and (d) With Na‐In‐S/Cu interlayer. (e) Adsorption energy model of Cu with PF_6_^−^ and (f) Na‐In‐S with PF_6_^−^, and (g) the corresponding comparison of adsorption energy. (h) AFM topography image of SEI about Cu, (i) Na‐In‐S/Cu, and (j) Comparison of the corresponding mechanical modulus. (k) Contour map of relaxation time distribution curves for the electrode with Na‐In‐S/Cu interlayer after 1 and 50 cycles. (l) Contour map of relaxation time distribution curves for the electrode without interlayer after 1 and 50 cycles.

To investigate the interfacial mechanism underlying the superior deposition behavior, we conducted a detailed compositional analysis of the SEI formed on with and without the Cu‐In‐S/Cu interlayer. XPS was employed to probe the SEI chemistry at different etching depths. For both samples (Figure 5a and Figure S16), the SEI showed similar compositions before etching, the C 1s spectrum exhibited a peak at 284.8 eV, attributed to C‐C/C‐H from solvent decomposition, and another at 289.5 eV, corresponding to Na_2_CO_3_ [44]. The F 1s spectrum in Figure 5a displayed a strong peak at 684.4 eV, corresponding to NaF [45]. After etching, the SEI formed with the interlayer exhibited a rapid decrease in organic components (C‐C, C‐O) with increasing depth, while the signals for Na and F in Figure 5a became more prominent. This indicates that the upper position of the SEI is organic rich, whereas inorganic species dominate the inner layer. In contrast, the SEI formed without the interlayer retained significant organic signals the entire depth (Figure S16). In addition, we also found that the SEI formed with the interlayer is much thinner and denser than that without the interlayer. On the electrode featuring Na‐In‐S/Cu interlayer, SEI signals nearly vanished at 100 nm, and In related signals appeared. In contrast, a distinct SEI signal is still detectable at a depth of 300 nm for the electrode without the interlayer, indicating that its SEI layer is significantly thicker than that of the former. (Figure 5b) [46].

Depth profiling by time‐of‐flight secondary ion mass spectrometry (TOF‐SIMS) further confirmed these findings. On the electrode without the interlayer, organic C_2_HO^−^ fragments were abundant at the surface and persisted with increasing etching depth (Figure 5c and Figure S17). In contrast, organic fragments were confined to the outermost layer and disappeared rapidly with depth, while inorganic fragments (NaF^−^) increased on the electrode featuring Na‐In‐S/Cu interlayer, confirming an inorganic dominated inner SEI (Figure 5d). Based on our calculations, these differences can be attributed to the strong Lewis acidity of In ions, which enhances PF_6_^−^ adsorption and promotes anion decomposition at the interface (Figure 5e–g), thereby facilitating the formation of an inorganic‐rich (NaF) SEI [37, 47]. Atomic force microscopy (AFM) analysis revealed the SEI on the electrode without the interlayer is rough (R_a_ = 20 nm) (Figure 5h), whereas the SEI on the electrode with the Na‐In‐S/Cu interlayer is thinner, denser (R_a_ = 9.4 nm) (Figure 5i), and mechanically stronger (Figure 5j). This robust inorganic‐rich SEI can better accommodates volume changes during cycling, suppresses dendrite growth, and enhance interfacial stability [48]. Further support for these observations was provided by DRT analysis, the electrode with Na‐In‐S/Cu interlayer remained stable after 50 cycles, indicating a consistent interfacial structure and efficient ion transport (Figure 5k). In contrast, the electrode without the interlayer exhibited increased peak intensity, reflecting higher impedance and ongoing side reactions (Figure 5l) [49].

### 2.5 Cycling Stability of Na‐In‐S/Cu Interlayer Based Na Metal Battery

**FIGURE 6:**
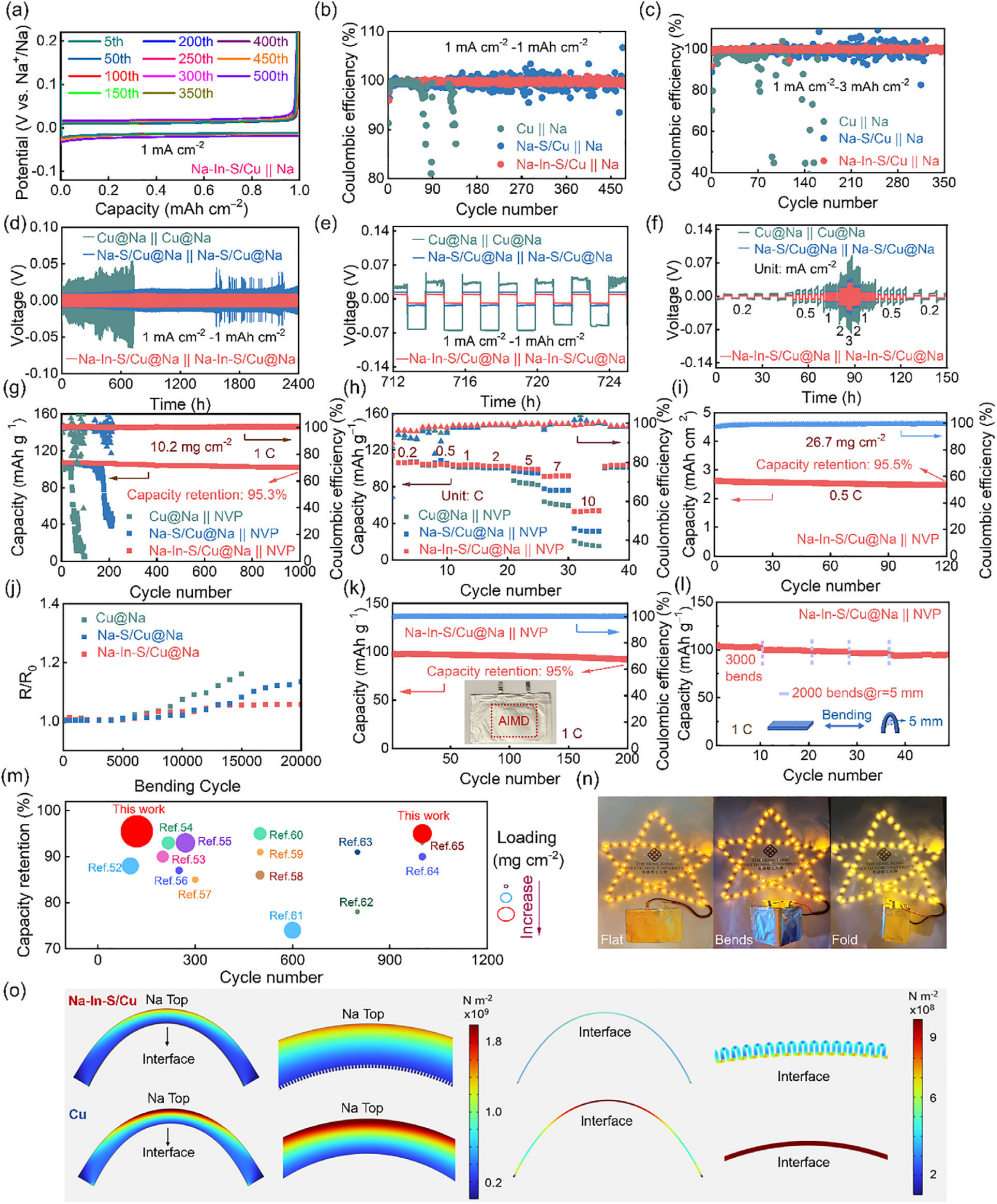
Electrochemical performance of different electrodes: (a) The GCD voltage profiles of the electrode featuring Na‐In‐S/Cu at the 5–500th cycles. CE of Cu||Na, Na‐S||Na and Na‐In‐S/Cu||Na cells at (b) 1.0 mA cm^−2^ for 1.0 mA h cm^−2^ and (c) 1.0 mA cm^−2^ for 3.0 mA h cm^−2^. (d) GCD voltage profiles in symmetric cells about Cu@Na||Cu@Na, Na‐S/Cu@Na||Na‐S/Cu@Na and Na‐In‐S/Cu@Na|| Na‐In‐S/Cu@Na cells at 1 mA cm^−2^ under 1 mA h cm^−2^, with additional details enlarged in (e). (f) Rate performance of various symmetric cells at current densities ranging from 0.2 to 3 mA cm^−2^. (g) Cycling stability of the full cells based on different electrodes featuring Cu@Na, Na‐S/Cu@Na, and Na‐In‐S/Cu@Na. (h) Rate performance of the full cells at high loading. (i) Cycling stability of ultra‐high loading NVP with Na‐In‐S/Cu@Na full cell. Flexibility demonstration: (j) Resistance changes of the electrode featuring Cu@Na, Na‐S/Cu@Na, and Na‐In‐S/Cu@Na as a function of bending cycles at a bending radius of 5 mm. (k) Cycling durability of the pouch cell of NVP// Na‐In‐S/Cu@Na. (l) Specific capacity of Na‐In‐S/Cu@Na//NVP pouch cells is recorded at 1 C for 50 cycles, in which 11,000 cycles of bending are performed with a small bending radius of 5 mm. (m) Corresponding performance comparison between our work and other reported. (n) Optical images of Na‐In‐S/Cu@Na//NVP pouch cell powering a large light‐emitting diode under original and various bending. (o) FEA of the bending behavior and strain distribution.

To systematically assess the electrochemical performance of Na plating/stripping on the 3D Na‐In‐S/Cu interlayer, electrochemical measurements were performed in a half‐cell. As illustrated in Figure 6a,b, the CE for Na plating/stripping on the electrode featuring Na‐In‐S/Cu interlayer was evaluated at a current density of 1 mA cm^−2^ with an area capacity of 1 mAh cm^−2^. The voltage‐capacity profiles of the electrode featuring Na‐In‐S/Cu interlayer across different cycles exhibit remarkable overlap, with only a slight increase in voltage hysteresis. Notably, the electrode featuring Na‐In‐S/Cu achieves an ultrahigh CE exceeding than 99.4% over approximately 500 cycles, demonstrating a highly reversible Na plating/stripping process. In contrast, the Cu fails to sustain normal Na plating/stripping over extended cycling. Its CE drops sharply to 59% after 80 cycles, followed by severe fluctuations, reflecting poor reversibility and uncontrolled interfacial reactions.

To further assess the stability of the electrode featuring Na‐In‐S/Cu interlayer under more practical conditions, the current density and areal capacity were increased to 1 mA cm^−2^–3 mAh cm^−2^ and 3 mA cm^−2^–3 mAh cm^−2^, respectively. As shown in Figure 6c, the electrode featuring Na‐In‐S/Cu interlayer maintains stable operation with high CE over 350 cycles at 1 mA cm^−2^–3 mAh cm^−2^. Even under elevated current density (3 mA cm^−2^) and high capacity (3 mAh cm^−2^), the CE remains more than 99% after 250 cycles (Figure S18). In contrast, the CE of the Cu become highly unstable under these conditions, reflecting aggravated interfacial side reactions and uncontrollable dendrite growth, these results demonstrate that the electrode featuring Na‐In‐S/Cu interlayer enables highly stable Na plating/stripping cycles across a wide range of deposition conditions.

To further investigate the long‐term cycling stability, symmetric cells were assembled using same Na predeposited electrodes, including Cu@Na, Na‐S/Cu@Na, and Na‐In‐S/Cu@Na. As shown in Figure 6d,e, the Cu@Na symmetric cells exhibited a rapid increase in overpotential shortly after the start of cycling, accompanied by visible voltage fluctuations, indicating poor stability. In comparison, the Na‐S/Cu@Na symmetric cells demonstrated improved cycling stability (Figure S19), however, significant voltage fluctuations still appeared after less than 1600 hours, suggesting limited long‐term durability. Remarkably, the introduction of In to form a stable, glue‐like interfacial layer in the Na‐In‐S/Cu@Na symmetric cells yielded a highly stable voltage profile and enabled an ultralong cycling lifespan of up to 2400 hours without short circuit. Furthermore, even at a high areal capacity of 3 mAh cm^−2^, the Na‐In‐S/Cu@Na symmetric cell maintained stable plating/stripping behavior for 1000 hours (Figure S20). These findings underscore the pivotal role of In induced interfacial engineering in enhancing the cycling stability and reversibility of Na metal electrodes. The rate performance of the symmetric cells was further evaluated at various current densities, as presented in Figure 6f. As the current density increased from 0.2 to 3 mA cm^−2^, the cycling stability of the electrode featuring bare Cu progressively deteriorated, accompanied by a marked increase in voltage fluctuations, which can be attributed to intensified interfacial side reactions at higher current densities. In contrast, the functionalized electrode featuring Na‐In‐S/Cu@Na exhibited highly stable voltage and maintained the lowest overpotential across all tested current densities, highlighting its superior rate capability and interfacial stability.

### 2.6 Full Cell Performance with High‐Mass‐Loading Cathodes

Full cells were assembled by pairing various electrodes with high‐mass‐loading NVP cathodes. When the NVP cathode was employed at a high mass loading of 10.2 mg cm^−2^, the electrode featuring the glue‐effect interlayer delivered a discharge capacity of 107 mAh g^−1^ and exhibited excellent capacity retention of 95.3% over 1000 cycles (Figure 6g). In contrast, the other two electrode demonstrated rapid capacity fading under high mass loading conditions. Remarkably, even at an elevated rate of 3 C, the electrode featuring glue‐effect interlayer maintained stable cycling with negligible capacity loss over nearly 300 cycles (Figure S21). Furthermore, rate capability tests revealed outstanding high‐rate performance, with the discharge capacity remaining nearly unchanged across a wide current density range from 0.2 to 7 C (Figure 6h). Notably, when the cathode mass loading was further increased to an ultrahigh level of 26.7 mg cm^−2^, the cell still retained an impressive 95.5% of its initial capacity after 120 cycles (Figure 6i), indicating excellent cycling stability even under extreme conditions.

To further assess the suitability of the electrode featuring Na‐In‐S/Cu interlayer for flexible electronics, we also conducted flexibility tests. The CuInS_2_ exhibited remarkable mechanical strength and flexibility. The tensile stress at break is approximately 129 MPa higher than that of commonly used flexible carbon cloth substrates (≈ 6 MPa) (Figure S22), and meets the requirements of industrial large‐scale production (>100 MPa) [50, 51]. Macroscopic observations confirmed that the electrode featuring Na‐In‐S/Cu interlayer could withstand complex deformations, including bending, folding, and twisting, further highlighting its robust mechanical flexibility (Figure S23). The electrode featuring Na‐In‐S/Cu@Na also demonstrated excellent flexibility, showing negligible resistance change after 20,000 bending cycles at a bending radius of 5 mm (Figure 6j).

To demonstrate the potential applications for wearable devices, flexible pouch cells were assembled using Na‐In‐S/Cu@Na anodes and high‐mass‐loading NVP cathodes (10.2 mg cm^−2^). The cells exhibited excellent cycling stability, retaining 95% of their capacity after 200 cycles with no distortion in the voltage profiles (Figure 6k and Figure S24), and maintained stable performance after 11,000 bending cycles at a 5 mm radius (Figure 6l), Figure S25 clearly illustrate the bending state of the pouch cell under different conditions. Compared to most reported literature [52, 53, 54, 55, 56, 57, 58, 59, 60, 61, 62, 63, 64, 65], our high‐mass‐loading cells exhibit superior cycling stability and areal capacity (Figure 6m and Table S1), indicating their strong potential for next‐generation Na batteries. Additionally, the pouch cells reliably powered LED displays under various bending conditions (Figure 6n), demonstrating their suitability for flexible and wearable electronics applications.

To gain deeper insight into the superior bending performance, finite element simulations were conducted to analyze the interfacial mechanics of Na deposition on both Cu and Na‐In‐S/Cu interlayer under bending conditions. Under the same displacement load, the simulation results reveal that the average interfacial stress on the Cu reaches 7.5 GPa, whereas the Na‐In‐S/Cu interlayer exhibits an average interfacial stress approximately 21% lower (Figure 6o). This reduction in stress is attributed to the sodiophilic nature and nanowalls structure of the glue effect stable Na‐In‐S/Cu interlayer, which increase the effective contact area and mitigate stress concentration at the interface [66]. Consequently, the Na‐In‐S/Cu@Na interface is less susceptible to fracture or delamination during bending, thereby maintaining robust electrical contact and delaying interfacial failure. These characteristics significantly enhance the mechanical flexibility and cycling reliability of the battery, making it particularly well suited for flexible electronic applications that demand repeated bending.

## 3 Conclusions

In conclusion, we report a 3D Cu/Na_5_InS_4_/Na_2_S composite interlayer that markedly enhances the cycling stability of Na metal anodes. This interlayer strengthens Na affinity and lowers the Na^+^ diffusion barrier, thereby reducing nucleation overpotential and facilitates rapid Na^+^ migration. Meanwhile, strong binding energy among the composite phases induces a “glue‐like” effect, ensuring robust cohesion and mechanical integrity. It also homogenizes the electric field and Na^+^ flux, suppressing local current hotspots and promoting uniform, dendrite‐free Na deposition. Benefiting from this intrinsically stable interphase, Na metal anodes featuring Na‐In‐S/Cu interlayer exhibit remarkable electrochemical performance, achieving an average CE exceeding 99.4% over 500 cycles in half cells, and 2400 h cycling in symmetric cells. SMBs equipping the composite interlayer demonstrate outstanding cycling stability with high‐mass‐loading NVP cathodes: they retain 95.3% capacity after 1000 cycles at a mass loading of 10.2 mg cm^−2^, and 95.5% capacity after 120 cycles at an ultrahigh mass loading of 26.7 mg cm^−2^. The high flexibility and adhesion of the composite interlayer also enable highly flexible SMB pouch cells that can readily pass10,000 bending test without obvious deterioration of the electrochemical properties.

This work presents an effective strategy for interfacial engineering through in situ formation of composite interfaces. This approach may be extended to other multicomponent precursors, such as sulfides, oxides, and selenides. Rationally selecting the precursors, it enables precise control over the metallic and ionic conductive phases, offering a pathway for constructing multiphase interlayers. The strategy is also applicable to various systems, including Li, Zn, and K. To gain a deeper understanding of the dynamic chemo‐mechanical evolution of these interfaces during cycling, integrating advanced operando characterization with multiscale modeling is expected to provide deeper mechanistic insights, thereby accelerating the development of next‐generation high‐energy‐density flexible energy storage systems.

In further enhancing the performance of Na metal batteries, high‐mass‐loading cathodes present a promising direction. The approach outlined in this work shows great potential in significantly increasing the battery capacity without compromising stability. Combining higher mass‐loading cathodes with robust interfacial engineering could pave the way for achieving higher capacity and longer lifespan in next‐generation Na metal batteries, thereby advancing efficient and durable energy storage solutions from the laboratory to practical applications.

## Conflicts of Interest

The authors declare no conflicts of interest.

## Supporting information




**Supporting File**: adma73556‐sup‐0001‐SuppMat.docx.

## Data Availability

The data that supports the findings of this study are available in the supplementary material of this article.
