# Supplementary material for: Indium‐Mediated Glue‐Like Interlayer Enables Stable High‐Capacity Flexible Sodium Metal Batteries
Source: Adv Mater. 2026 May 29;38(37):e73556. doi: 10.1002/adma.73556 (PMC13327209; doi:10.1002/adma.73556)
Supplement: Supplementary file 1 — Supporting File: adma73556‐sup‐0001‐SuppMat.docx. [file ADMA-38-e73556-s001.docx]

Supporting Information

Indium-mediated Glue-like Interlayer Enables Stable High-Capacity Flexible Sodium Metal Batteries

Xinyan Li, Shujing Wen, Junhua Zhou, Yanpeng Guo, Jiehua Cai, Xingyang Wang, Yongqiang Yang, Ang Ye, Can Guo, Zhaokun Wang, Qiyao Huang, M. Danny Gu, John Wang, Zijian Zheng*

Dr. X. Li, S. Wen, Dr. J. Zhou, Dr. Y, Guo, Dr. J. Cai, Dr. Y. Yang, Dr. C. Guo, Z. Wang, Prof. Z. J. Zheng

Department of Applied Biology and Chemical Technology, Faculty of Science, The Hong Kong Polytechnic University, Hung Hom, Hong Kong SAR, 999077, China

E-mail: tczzheng@polyu.edu.hk (Z. J. Zheng)

Prof. Z. J. Zheng

Research Institute for Smart Energy, The Hong Kong Polytechnic University, Hung Hom, Hong Kong SAR, 999077, China

Research Institute for Intelligent Wearable Systems, The Hong Kong Polytechnic University, Hung Hom, Hong Kong SAR, 999077, China

PolyU-Daya Bay Technology and Innovation Research Institute, Huizhou, Guangdong, 516083, China

PolyU-Wenzhou Technology and Innovation Research Institute, Wenzhou, Zhejiang, 325024, China

A. Ye, Dr. Q. Huang

School of Fashion and Textiles, The Hong Kong Polytechnic University, Hong Kong SAR, 999077, China

X. Wang, Prof. J. Wang

Department of Materials Science and Engineering, National University of Singapore, Singapore, 117574, Republic of Singapore

Prof. M. D. Gu

Institute for Advanced Study, Eastern Institute of Technology, Ningbo, Zhejiang, 315200, China

**Method**

*Finite-element Analysis:* We employed finite element simulation software to investigate the growth process of Na dendrites on the electrode surface. The simulation utilized the tertiary current distribution (Nernst-Planck) interface and the level set interface to model the Na electrode reaction and the Na deposition process, respectively. The initial Na concentration in the electrolyte was set to 1 M, and the exchange current density of the electrode was set to 0.5 mA cm^−2^. The level set interface was used to track the morphological evolution of dendrites induced by Na ion deposition. In the simulation, the electrode boundary with various morphologies at the bottom was defined as the level set inlet, while the top boundary served as the outlet. The level set method was employed to capture the dynamic changes of the dendrite-electrode interface, with the interface velocity proportional to the local current density of the electrode reaction, expressed as $Vₙ =\frac{I}{MF\rho}$, where I is the local current density, M is the molar mass of Na, ρ is the density of Na, and F is the Faraday constant. Transient solvers were used to resolve the temporal evolution of electrolyte current, concentration, and dendritic electrode morphology. To simplify the computation, a two-dimensional simulation model was constructed*.*

The solid mechanics module was employed to simulate and analyze the stress distribution of two different structures under bending loads. The geometric model was constructed as a two-dimensional plane stress structure, with the centerline defined as the axis of symmetry. To induce controlled bending deformation, equal-magnitude but opposite-direction horizontal displacement loads (5 μm, 10 μm, and 20 μm) were applied to the left and right endpoints at the bottom of the model. This approach enabled the investigation of stress distributions in the samples under varying degrees of bending.

*Density Functional Theory (DFT)* *Calculation:* The Perdew-Burke-Ernzerhof (PBE) functional under the generalized gradient approximation (GGA) was employed to account for electron exchange and correlation effects^[1-3]^ A plane-wave cutoff energy of 450 eV was used for the expansion of electronic wave functions. To eliminate interactions between periodic images, a vacuum layer of 15 Å was introduced along the z-axis. Structural optimizations were conducted until the total energy converged to 1×10^–5^ eV. The adsorption energy of Na (E_ads_) was determined using the following expression:

E_ads_ = E_total_ – E_base_ -E_Na_

where E_total_ is the total energy of the substrate with an adsorbed Na atom, and E_base_ and E_Na_ denote the energies of the clean substrate and an isolated Na atom, respectively.

.

Experimental Section

*Preparation of GF@Cu:* Cu was deposited onto commercial glass fiber (GF) via a polymer-assisted metal deposition (PAMD) method. In brief, the GF was first immersed in a silanization solution comprising 1% (v/v) acetic acid, 4% (v/v) MPTS, 4% (v/v) deionized water, and 95% (v/v) ethanol for 60 min at room temperature. Following silanization, the GF was thoroughly rinsed with deionized water and then placed in a 20% METAC aqueous solution containing potassium persulfate (2 g L^−1^) as the initiator, with polymerization carried out at 85 °C for 60 min. After additional rinsing with deionized water, the polymer-coated GF was immersed in a 5 mM (NH_4_)_2_PdCl_4_ solution for 60 min to enable ion exchange with the [PdCl_4_]^2-^ catalyst. The catalyst-loaded GF was washed several times and then subjected to electroless Cu deposition to form a thin Cu layer on the surface. The deposition bath consisted of a 1:1 (v/v) mixture of two solutions: Solution A contained CuSO_4_·5H_2_O (13 g L^−1^), NaOH (12 g L^−1^), and KNaC_4_H_4_O_6_·4H_2_O (29 g L^−1^) in deionized water, while Solution B was an aqueous formaldehyde solution (9.5 mL L^−1^). The ELD process was conducted for 20 minutes.

*Preparation of CuInS_2_*: 1 mmol copper(I) chloride, 1 mmol indium(III) chloride tetrahydrate, and 3 mmol thiourea were dissolved in deionized water under continuous stirring until a clear and transparent solution was obtained. This solution was then transferred to a 50 mL Teflon-lined stainless steel autoclave containing Cu-GF fibers, and subjected to hydrothermal treatment at 160 °C for 8 hours. After the reaction, the fibers were removed and thoroughly washed several times with ethanol and deionized water. The final CuInS_2_ product was obtained by annealing at 300 °C under an argon atmosphere for 2 hours. For the synthesis of CuS, the same procedure was followed, except indium(III) chloride tetrahydrate was omitted from the reaction mixture.

*Material characterization:* The morphology of all samples was examined using field emission scanning electron microscopy (FESEM, Tescan Vega3) and transmission electron microscopy (TEM, JEOL-2100F). Scanning transmission electron microscopy (STEM) analysis was carried out on a probe aberration-corrected TEM (Thermofisher Spectra 300) operated at 200 kV. The crystal structures were identified by X-ray diffraction (XRD, Bruker D8). The interfacial chemical composition was analyzed via X-ray photoelectron spectroscopy (XPS, PHI 5000 Versaprobe Ⅲ) equipped with a monochromatic Al K𝛼 source. Mechanical properties were evaluated by recording stress–strain curves using a universal testing machine (Instron 5565A tester).

*Electrochemical measurement:* Electrochemical measurement: Coulombic efficiency was evaluated for Cu, Na-S/Cu, and Na-In-S/Cu||Na half cells, respectively. Symmetric cells comprising Cu@Na, Na-S/Cu@Na, and Na-In-S/Cu@Na electrodes were assembled to assess long-term cycling stability. For full cell tests, Cu@Na, Na-S/Cu@Na, and Na-In-S/Cu@Na served as the anodes, while the cathode consisted of a mixture containing 90% Na_3_V_2_(PO_4_)_3_, 5% carbon black, and 5% polyvinylidene fluoride. In pouch cell fabrication, Na_3_V_2_(PO_4_)_3_ was used as the cathode and Na-In-S/Cu@Na as the anode, with the cell sealed in an aluminum laminate composite foil. The electrolyte employed was 1 M NaPF_6_ dissolved in Diglyme. Electrochemical impedance spectroscopy was performed over a frequency range of 100 kHz to 0.1 Hz with an applied voltage of 5 mV using an EC-Lab workstation (Biologic VMP3, France). Sodium electrochemical performance was measured on a Neware battery testing system (CT2001A).

**Figure S1.** Morphology and compositional Cu sample: a-c) SEM image of top views. d-f) Element mapping, showing the compositional evenly distributed.

**Figure S2.** XPS core-level spectra of Cu 2p.

**Figure S3.** Morphology and compositional CuInS_2_ sample: a-d) SEM element mapping of top views. e-h) TEM element mapping, showing the Cu, In and S compositional uniform distributed.

**Figure S4**. Synthesis and characterizations of the CuInS_2_ nanowalls: a) high-resolution TEM image, and b) selected-area electron diffraction.

**Figure S5**. a) *In situ* EIS spectrum changes during the initial discharge before plating. b) Distribution of relaxation time curves of CuInS_2_.

**Figure R6**. The potential-current curves at a scan rate of 0.2 mV s^−1^.

**Figure S7.** Electrochemical performance of the different electrodes. Sodiophilic reaction (light-orange region region) and Na plating (light-green) illustrated by corresponding voltage profiles and schematics.

**Figure S8**. The capacity-voltage curves of half-cells at 1mA cm^−2^ for 1 mAh cm^−2^. a-b) Na-In-S/Cu||Na. c-d) Na-S/Cu||Na.

**Figure S9**. *In situ* XRD after cycling of the electrode featuring Na-In-S/Cu interlayer.

**Figure S10**. Geometrically optimized models and corresponding adsorption energy among CuS and CuInS_2_ with Na.

**Figure S11**. Simulation results for Na-In-S/Cu interlayer and Cu of electric field distribution.

**Figure S12**. Demonstration SEM images of the Cu after Na plating with deposition amount controlled as 3 mAh cm^−2^.

**Figure S13**. Demonstration SEM images of the electrode featuring Na-S/Cu after Na plating with deposition amount controlled as 3 mAh cm^−2^.

**Figure S14**. Demonstration SEM images of the electrode featuring Na-In-S/Cu after Na plating with deposition amount controlled as 3 mAh cm^−2^.

**Figure S15**. a) Side-views SEM images of the electrode featuring Na-In-S/Cu interlayer and b) Cu after cycles at plating.

**Figure S16**. Characterization on the surface chemistry of the Cu after plating and stripping: High resolution XPS spectra of the SEI film at different etching depth.

**Figure S17**. Depth distribution profiles of various ions as a function of sputtering time.

**Figure S18**. Coulombic efficiency comparison of the Cu||Na, Na-S/Cu||Na, and Na-In-S/Cu||Na half-cells for 3 mAh cm^−2^ at current density of 3 mA cm^−2^.

**Figure S19**. Galvanostatic plating/stripping profiles of symmetric cells on the different electrodes at 1 mA cm^−2^ for 1 mAh cm^−2^.

**Figure S20**. Galvanostatic plating/stripping profiles of symmetric cells on the electrode featuring Na-In-S/Cu@Na at 1 mA cm^−2^ for 3 mAh cm^−2^.

**Figure S21**. Cycling performance of full cells at 3 C.

**Figure S22**. Comparison in tensile strain and stress of different materials.

**Figure S23**. Demonstration of the flexibility of the a-d) CuInS_2_ and e-h) Na-In-S/Cu@Na. The composite can be arbitrarily scrolled, flexed, twisted and folded without the obvious creeping.

**Figure S24**. Charge-discharge curves of Na-In-S/Cu@Na||Na_3_V_2_(PO_4_)_3_ full cells at 1 C in different cycles.

**Figure S25**. The optical images of pouch cells during different bending states.

**Table S1.** Corresponding performance comparison between our work and other reported

| Sample | Capacity retention | Loading mg cm^-2^ | Reference |
| --- | --- | --- | --- |
| NVP\|\|Sb@HPCNF@Na | 1000 cycles with 93% | 1 | ^[4]^ |
| NVP\|\|PPNM@Na | 800 cycles with 78.2% | 1.5-2 | ^[5]^ |
| NVP\|\|Sb_2_S_3_-CNFs@Na | 800 cycles with 91.0% | 1.8-2.0 | ^[6]^ |
| Sb-CuF_2_@Cu/Na | 400 cycles with 87% | 2 | ^[7]^ |
| NVP\|\|MRS800-ZnSA@Na | 300 cycles with 85.0% | 3 | ^[8]^ |
| NVP\|\|AAS\|\|HC@Na | 500 cycles with 91.3% | 3 | ^[9]^ |
| Na_3_V_2_(PO_4_)_2_F_3_\|\|Na | 250 cycles with 87.1% | 4 | ^[10]^ |
| NVP\|\|Sn_4_P_3_-NPGTS@Na | 1000 cycles with 90.8% | 4 | ^[11]^ |
| NVP\|\|BEOP50%\|\|Na | 500 cycles with 86.2% | 5.7 | ^[12]^ |
| NVP\|\|CNT@Na | 270 cycles with 93.9% | 12 | ^[13]^ |
| NVP\|\|SnCu@Al@Na | 400 cycles with 93.0%  200 cycles with 90.0% | 4.5  7.6 | ^[14]^ |
| NVP\|\|Na | 500 cycles with 95.9% | 8 | ^[15]^ |
| NVP\|\|Na | 217 cycles with 93.5% | 8.8 | ^[16]^ |
| NVP\|\|Cu_2_Sb@Cu | 600 cycles with 74.2% | 10 | ^[17]^ |
| NVP\|\|Ag@Na | 100 cycles with 88.0% | 10 | ^[18]^ |
| NVP\|\| Na-In-S/Cu@Na | 1000 cycles with 95.3%  120 cycles with 95.5% | 10  26.7 | This work |

**Reference**

[1]	P. E. Blochl, *Phys. Rev. B,* **1994**, 50, 17953.

[2]	G. Kresse, J. Furthmüller, *Phys. Rev. B,* **1996**, 54, 11169.

[3]	J. Perdew, K. Burke, M. Ernzerhof, *Phys. Rev. Lett.,* **1996**, 77, 3865.

[4]	Z. Li, H. Qin, W. Tian, L. Miao, K. Cao, Y. Si, H. Li, Q. Wang, L. Jiao, *Adv. Funct. Mater.,* **2023**, 34, 2301554.

[5]	W. Tian, G. Lin, S. Yuan, T. Jin, Q. Wang, L. Jiao, *Angew. Chem. Int. Ed.,* **2025**, 64, 202423075.

[6]	X. Lu, R. Chen, S. Shen, Y. Li, H. Zhao, H. Wang, T. Wu, Y. Su, J. Luo, X. Hu, S. Ding, W. Li, *Nano Lett.,* **2024**, 24, 5490.

[7]	X. Zheng, Z. Zhang, Z. Li, C. Shi, Y. Yamauchi, J. Tang, *Nano Energy,* **2025**, 138, 110858.

[8]	S. Zhao, X. Chen, D. Patrun, L. Zheng, L. Wang, Z. Wen, S. Mathur, Z. Hong, *Small,* **2025**, 21, 2410941.

[9]	X. Li, T. Zhang, Y. Zhao, X. Zhu, A. Ge, K. C. Gordon, F. Wang, G. Xu, M. Zhu, *J. Am. Chem. Soc.,* **2025**, 147, 8488.

[10]	Y. Wang, H. Yang, J. Xu, P. Tang, Q. Wei, T. Hu, X. Gao, Z. Guo, R. Fang, G. Hu, S. Bai, F. Li, *J Am Chem Soc,* **2024**, 146, 7332.

[11]	H. Zong, S.-Q. Li, X. Zheng, X.-Y. Wu, S. S. Li, Qian-Qian Hao, X.-H. Zhao, Y.-W. Zhang, K.-X. Wang, X. Song, J.-S. Chen, *J. Am. Chem. Soc.,* **2025**, 147, 29779.

[12]	M. Wu, M. Yang, J. Yu, X. Ma, S. Sun, Y. She, J. Yang, X. Zou, Y. Hu, F. Yan, *J. Am. Chem. Soc.,* **2024**, 146, 35229.

[13]	H. Wang, J. Wang, W. Li, J. Hu, J. Dong, D. Zhai, F. Kang, *Adv. Mater.,* **2024**, 36, 2409062.

[14]	J. Shi, D. Wang, Q. Liu, Z. Yu, J.-Q. Huang, B. Zhang, *Nano Lett.,* **2025**, 25, 1800.

[15]	W. Chen, J.-S. Park, C. Kwon, C. O. Plaza-Rivera, C.-W. Hsu, J. K. Phong, L. J. Kilgallon, D. Wang, T. Dai, S. Y. Kim, G. Zhu, Y. Gao, Z. Ren, Z. Zhang, H. Lim, Y. Shao-Horn, J. A. Johnson, J. Li, *Joule,* **2025**, 9, 101811.

[16]	T. Shi, R. Hou, L. Zheng, H. Lu, C. Xu, X. Sun, P. He, S. Li, H. Zhou, S. Guo, *Adv. Energy Mater.,* **2025**, 15, 2405803.

[17]	C. Xie, H. Wu, J. Dai, Z. Fu, R. Zhang, H. Ji, Q. Zhang, Y. Tang, T. Qiu, H. Wang, *Adv. Energy Mater.,* **2024**, 14, 2400367.

[18]	W. Zhang, Q. Lu, G. Sun, Z. Chen, P. Yue, G. Zhang, B. Song, K. Song, *Nano Lett.,* **2025**, 25, 6092.
